# Langerhans cell precursors acquire RANK/CD265 in prenatal human skin

**DOI:** 10.1016/j.acthis.2015.01.003

**Published:** 2015

**Authors:** Alice Schöppl, Albert Botta, Marion Prior, Johnnie Akgün, Christopher Schuster, Adelheid Elbe-Bürger

**Affiliations:** a Department of Dermatology, Division of Immunology, Allergy and Infectious Diseases (DIAID), Laboratory of Cellular and Molecular Immunobiology of the Skin, Medical University of Vienna, Vienna, Austria; b Institute of Specific Prophylaxis and Tropical Medicine, Medical University of Vienna, Vienna, Austria

**Keywords:** BMP7, bone morphogenetic protein 7, CCL5, chemokine (C–C motif) ligand 5, DC, dendritic cell, EGA, estimated gestational age, GM–CSF, granulocyte–macrophage colony-stimulating factor, LC, Langerhans cell, RANK(L), receptor activator of nuclear factor-kappaB (ligand), SCF, stem cell factor, TGF-β, transforming growth factor beta, TNF, tumor necrosis factor, T_reg_, regulatory T cell, Prenatal, Skin, Development, RANK, Langerhans cells, Precursor

## Abstract

The skin is the first barrier against foreign pathogens and the prenatal formation of a strong network of various innate and adaptive cells is required to protect the newborn from perinatal infections. While many studies about the immune system in healthy and diseased adult human skin exist, our knowledge about the cutaneous prenatal/developing immune system and especially about the phenotype and function of antigen-presenting cells such as epidermal Langerhans cells (LCs) in human skin is still scarce. It has been shown previously that LCs in healthy adult human skin express receptor activator of NF-κB (RANK), an important molecule prolonging their survival. In this study, we investigated at which developmental stage LCs acquire this important molecule. Immunofluorescence double-labeling of cryostat sections revealed that LC precursors in prenatal human skin either do not yet [10–11 weeks of estimated gestational age (EGA)] or only faintly (13–15 weeks EGA) express RANK. LCs express RANK at levels comparable to adult LCs by the end of the second trimester. Comparable with adult skin, dermal antigen-presenting cells at no gestational age express this marker. These findings indicate that epidermal leukocytes gradually acquire RANK during gestation – a phenomenon previously observed also for other markers on LCs in prenatal human skin.

## Introduction

The receptor activator of nuclear factor-kappaB ligand (RANKL/CD254), its signaling receptor RANK/CD265 and the decoy receptor osteoprotegerin are members of the tumor necrosis factor (TNF) and TNF-receptor superfamily (Sigl and Penninger, 2014). RANKL is found on osteoblasts, basophils, activated keratinocytes and T cells, while RANK is constitutively expressed in osteoclasts and dendritic cells (DCs) (Barbaroux et al., 2008; Huber et al., 2014; Kukita and Kukita, 2013; Leibbrandt and Penninger, 2008; Loser and Beissert, 2007). RANKL/RANK have multiple functions ranging from bone physiology, mammary gland formation, lymph node development, initiation of breast cancer and immune regulation (Arizon et al., 2012; Beristain et al., 2012; Hanada et al., 2011; Kukita and Kukita, 2013; Loser and Beissert, 2007; Sigl and Penninger, 2014).

Langerhans cells (LCs) represent the DC subset in the epidermis and other stratified epithelia. Under steady-state conditions LCs have been implicated to maintain tolerance (Seneschal et al., 2012). Upon infectious challenge they can drive T helper (T_H_) T_H_1, T_H_2 (Furio et al., 2010), T_H_17 (Mathers et al., 2009) and T_H_22 (Fujita et al., 2009) responses. In healthy adult human epidermis, ∼95% of LCs express RANK when analyzed by flow cytometry (Barbaroux et al., 2008) while keratinocytes express low levels of RANKL. Upon ultraviolet irradiation or infection of the skin in mice, keratinocytes upregulate RANKL and trigger RANK expressing LCs (Loser et al., 2006; Yamaguchi and Sakaguchi, 2006). The activated LCs produce cytokines [interleukin (IL)-6, IL-10, TNF-α], upregulate costimulatory molecules (CD86, CD205) and preferentially induce the proliferation of CD4^+^CD25^+^ regulatory T cells (T_reg_) which are able to suppress local and systemic immune reactions (Loser et al., 2006). Similarly, transgenic mice expressing RANKL under the keratinocyte-specific K14 promotor have increased T_reg_ numbers in peripheral lymphoid organs which can subsequently reduce and delay the onset of CD40L-induced autoimmune dermatitis in mice (Loser et al., 2006). Depletion of RANKL in mice results in a decrease of LC numbers as well as their proliferation rate without affecting morphology (Barbaroux et al., 2008).

During prenatal human skin development, LC precursors start to populate the epidermis at 7–8 weeks estimated gestational age (EGA). The LC phenotype is acquired in a step-wise manner starting with human leukocyte antigen (HLA) class II followed by CD1c, CD207 and CD1a (Elbe-Bürger and Schuster, 2010; Foster et al., 1986; Fujita et al., 1991; Schuster et al., 2009, 2014). At 18 weeks of gestation, the marker profile is similar to that of adult LCs expressing CD1a, CD1c, CD39, CD207, CCR6, CD324, and HLA class I and II (Elbe-Bürger and Schuster, 2010). The purpose of this study was to examine at which developmental stage LCs acquire RANK expression.

## Materials and methods

Embryonic and fetal human skin was obtained from legal abortions, ranging between 10 and 23 weeks EGA (*n* = 12). Healthy adult human breast and belly skin (25–60 years; *n* = 7) was obtained after plastic surgery. The study was approved by the local ethics committee and conducted in accordance with the Declaration of Helsinki principles. The parents and adult volunteers were informed and gave their written permission.

Skin specimens were cut into small pieces, embedded in Tissue-Tek^®^ optimum cutting temperature formulation compound, snap-frozen in liquid nitrogen and stored at −80 °C. Frozen sections (5 μm) of skin were cut, fixed in ice-cold acetone, air-dried and stained.

To prepare epidermal sheets from adult human skin, the subcutaneous tissue was removed, the remaining skin cut into small pieces (∼1 cm^2^) and floated dermal side down on a 3.8% ammonium thiocyanate solution (Merck, Darmstadt) (1 h, 37 °C). Subsequently, epidermal sheets were carefully peeled from the dermis using forceps, washed with phosphate-buffered saline (pH 7.4; 2× 5 min each), fixed with acetone (10 min, room temperature), washed again and either stained immediately as described below or stored in Eppendorf tubes at −80 °C until use.

Skin sections and epidermal sheets were incubated with an unconjugated anti-RANK mAb (clone 80704; R&D Systems, Minneapolis, MN) in 2% bovine serum albumin/phosphate-buffered saline and subsequently stained with an Alexa Fluor 546-labeled reagent (Invitrogen, San Diego, CA). In the next step mAbs against either HLA-DR (clone L243; FITC-labeled, BD Pharmingen, San Diego, CA) or CD1a (clone HI149; Alexa Fluor 488-labeled, Biolegend, San Diego, CA) were applied. Finally, the samples were washed twice and a nuclear staining was performed with Hoechst dye (Invitrogen). Control samples were stained with appropriate isotype-matched Ab. HLA-DR^+^RANK^+^ and HLA-DR^+^RANK^−^ cells in human epidermis have been enumerated on cryostat sections and placed in relation to the length of the epidermis (Olympus AX70, Olympus Corp., Tokyo). Single and double positive cells were counted in cryosections and epidermal sheets. Differences between groups were assessed with Student's *t*-test (GraphPad Software, San Diego, CA). The reported *p*-value is a result of a two-sided test. A *p*-value <5% is considered statistically significant.

## Results

### The majority of LCs in adult human epidermis express RANK

**Fig. 1:**
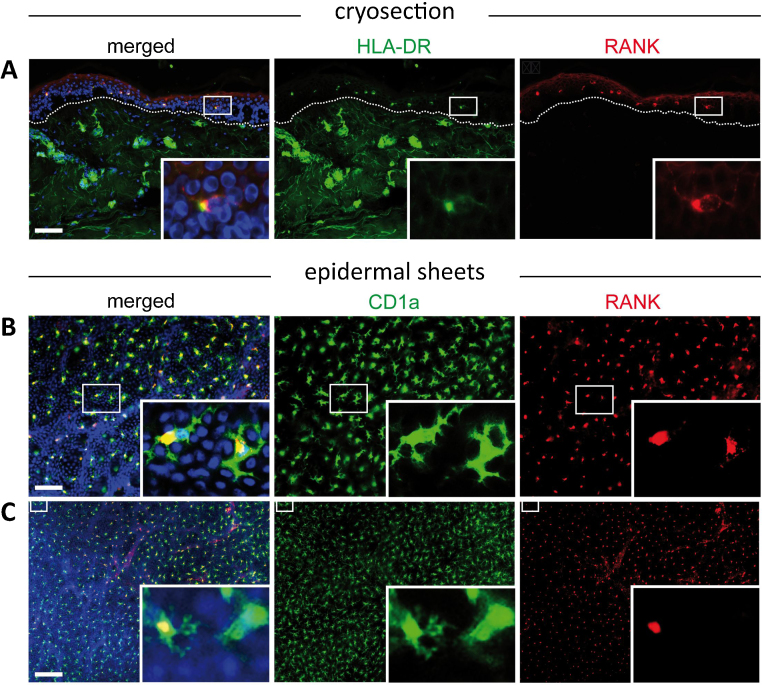
Not all LCs express RANK in adult human skin in situ. Immunofluorescence double-labeling was performed on cryostat sections of adult human skin and revealed the presence of HLA-DR^+^RANK^+^ LCs in the epidermis (insert) and HLA-DR^+^RANK^−^ cells in the dermis (upper panel). One representative donor out of seven is shown. Double immunofluorescence labeling of epidermal sheets showed that most, but not all, CD1a^+^ epidermal cells co-express RANK (middle and lower panel). Shown is one representative donor out of seven. Nuclei were stained with Hoechst dye. Scale bars: 50 μm (upper and middle panel), 100 μm (lower panel).

Double immunofluorescence staining of healthy adult human skin using cryostat sections and epidermal sheets revealed that LCs co-express HLA-DR, the CD1a and RANK (Fig. 1A–C) confirming previously reported results (Barbaroux et al., 2008). Whereas HLA-DR is expressed on epidermal LCs, dermal antigen-presenting cells and endothelial cells, RANK expression in adult skin is confined to LCs. Based on previous findings which report that not all LCs express RANK when evaluating single cell suspensions by flow cytometry (Barbaroux et al., 2008), it was our goal to investigate whether it is possible to detect CD1a^+^RANK^−^ cells in situ as well. We employed the epidermal sheet staining method as it allows en face view of the whole LC network in a given area compared to the few LCs in a cryostat section. When comparatively analyzing skin from different body regions, we found that the great majority of LCs expresses both CD1a and RANK. In addition, we always detected a small but distinct population of CD1a^+^RANK^−^ cells with a dendritic morphology (Fig. 1C, insert), supplementing previously reported flow cytometry data (Barbaroux et al., 2008). In contrast to CD1a, RANK expression is essentially confined to the perinuclear cytoplasm and is consequently only faintly or not expressed on dendrites. Furthermore, we found that RANK is weakly expressed in some LCs implying either a continuum of RANK^low^ to RANK^high^ or vice versa in adult skin (Fig. 1A,B). When enumerating CD1a^+^RANK^−^ LCs in epidermal sheets, we found that a comparable percentage of CD1a^+^ LCs in breast, back, and abdominal skin failed to express RANK, indicating that CD1a^+^RANK^−^ cells are similarly distributed in the analyzed body locations (data not shown).

### LC precursors in prenatal human skin acquire RANK in a gradual manner

**Fig. 2:**
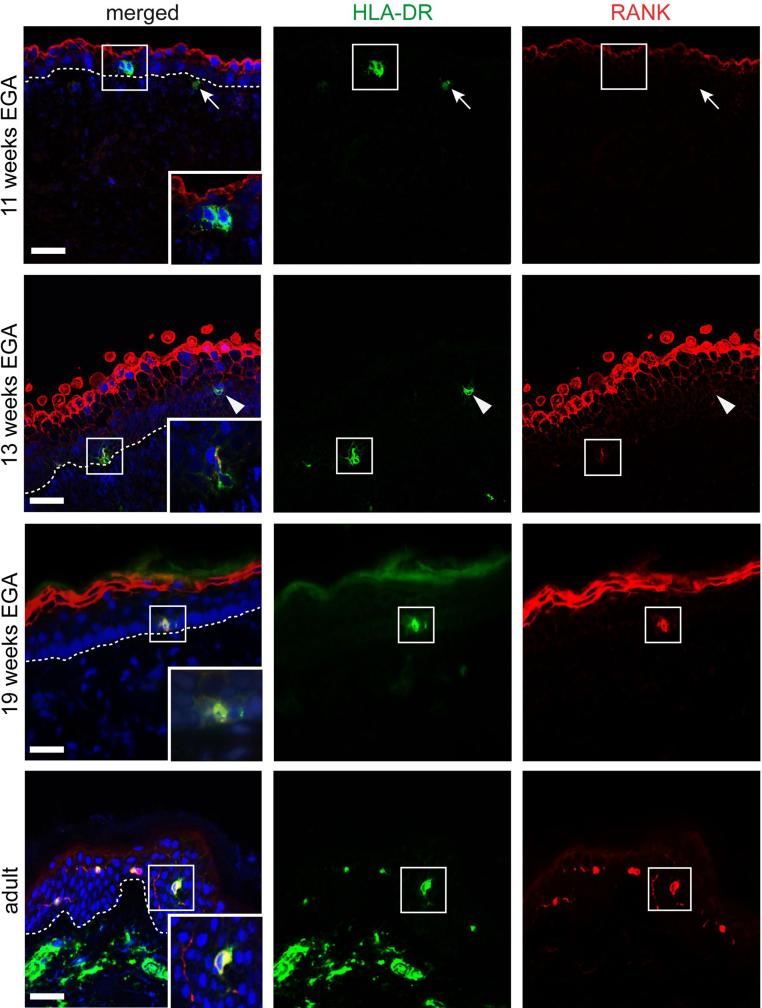
RANK is acquired in a stepwise manner during prenatal skin development. Immunofluorescence double-labeling was performed on cryostat sections of embryonic, fetal and adult human skin. Inserts indicate the acquisition of RANK expression on HLA-DR^+^ epidermal leukocytes during gestation compared to adult skin. Arrows mark HLA-DR^+^RANK^−^ cells in the dermis at 11 weeks EGA. Arrowheads indicate HLA-DR^+^RANK^−^ cells in the epidermis at 13 weeks EGA, a time point when HLA-DR^+^RANK^low^ cells (insert) start to appear in the epidermis. The staining intensity of RANK^+^ epidermal leukocytes at 19 weeks EGA is comparable to those observed in adult human skin. At least three donors per age group were analyzed. The dotted lines represent the dermal–epidermal junction. Scale bars: 50 μm.

**Fig. 3:**
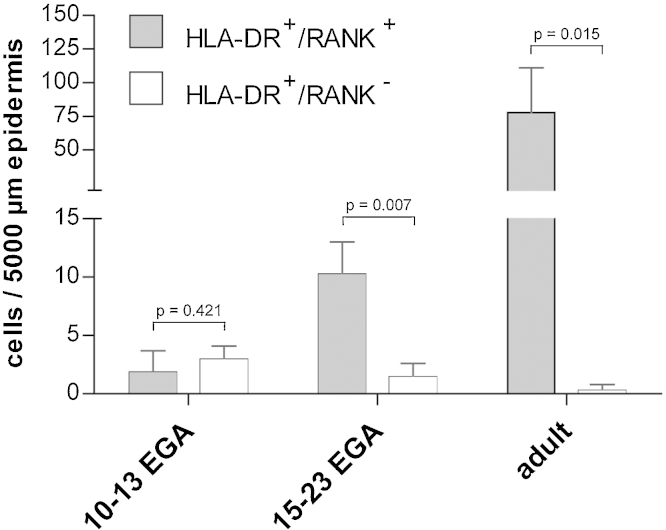
RANK expression on LC precursors arises early during skin development. The numbers of HLA-DR^+^RANK^+^ and HLA-DR^+^RANK^−^ cells in human prenatal epidermis have been enumerated on cryostat sections of indicated age groups. Bars represent the mean of investigated groups (*n* = 3 per group).

As RANK is (i) an important LC survival factor, (ii) a mediator for cell differentiation and (iii) not expressed in all LCs in adult epidermis (Fig. 1 and Barbaroux et al., 2008), we investigated its expression in human prenatal skin to evaluate when LC precursors start to express this molecule. HLA-DR is the first antigen-presenting cell specific marker to be found in embryonic human skin (Foster et al., 1986 Schuster et al., 2009), and was therefore employed to identify leukocyte precursors in prenatal skin. As shown in Fig. 2, double immunofluorescence staining of cryostat sections confirmed the presence of some rare HLA-DR^+^ epidermal (insert) and dermal (arrow) leukocytes none of which express RANK at 10 and 11 weeks EGA. At 13 weeks EGA, we found that some but not all HLA-DR^+^ cells in the epidermis express low levels of RANK (Fig. 2, arrow and insert). Surprisingly, we detected strong RANK expression in the periderm – a development-specific cell layer – at this time point of gestation (Fig. 2). With advancing gestational age, we found a spectrum of RANK^low–high^ leukocytes (data not shown) reaching adult-like staining intensity levels at 19–23 weeks EGA (Fig. 2, insert) while the density of RANK^+^ cells in fetal epidermis is not yet comparable to that in adults (10 vs. 75 cells/5000 μm epidermis) (Fig. 3). HLA-DR^+^ dermal cells at no time point of investigation express RANK. Statistical analysis revealed that in first trimester skin 67.7% [standard deviation (SD) 28.0%, *n* = 3] of all HLA-DR^+^ epidermal cells lack expression of RANK. The frequency of HLA-DR^+^RANK^−^ epidermal cells decreases with gestational age to 13.0% (SD 12.1%, *n* = 3) in second trimester skin and to 0.2% in adult skin (SD 0.46%; *n* = 3) (Fig. 3).

## Discussion

In the current study we provide evidence that LCs undergo a transition regarding the expression of RANK in healthy skin during gestation. We found a major population of HLA-DR^+^RANK^−^ epidermal leukocytes and a minor population of HLA-DR^+^RANK^+^ cells in embryonic epidermis. Later during development, RANK^+^ epidermal leukocytes outnumber RANK^−^ cells. We also identified a small population of CD1a^+^RANK^−^ LCs in adult skin in situ supplementing published flow cytometry data (Barbaroux et al., 2008). However, it is currently unclear whether these cells represent either RANK^−^ LC precursors or LCs that have downregulated RANK. In addition, also CD1a^−^RANK^+^ epidermal cells were described (Barbaroux et al., 2008). Their further characterization in our laboratory revealed their keratinocyte nature. Moreover we found that neither T cells nor melanocytes express RANK (Akgün, 2010).

In prenatal human skin, the leukocyte network has to develop early to ensure protection from the hostile milieu after birth (Schuster et al., 2012). Indeed, LC and dermal DC precursors populate the skin early during gestation and at around 18 weeks of EGA they already show the same marker profile and are morphologically similar to adult LCs (Elbe-Bürger and Schuster, 2010 Foster et al., 1986 Schuster et al., 2009). Key cytokines required for LC development, such as bone morphogenetic protein 7 (BMP7) and transforming growth factor beta-1 (TGF-β1) are detectable in the epidermis by 8 and 9 weeks EGA, respectively. Both molecules show an inverse expression pattern in the stratified epidermis: BMP7 in the basal layer and TGF-β1 exclusively in the suprabasal layer (Elbe-Bürger and Schuster, 2010 Yasmin et al., 2013). During maturation of the epidermis, TGF-β1 production precedes the acquisition of markers like Lag CD207 and CD1a on LC precursors at 12–13 weeks EGA (Schuster et al., 2009). Interestingly, this also correlates with our findings that RANK expression commences at around 13 weeks EGA, at the same time point when TGF-β production starts to increase (Schuster et al., 2009). Furthermore, higher concentrations of proliferation-inducing cytokines like IL-6, chemokine (C–C motif) ligand 5 (CCL5), stem cell factor (SCF) and granulocyte–macrophage colony-stimulating factor (GM-CSF) are present in prenatal skin cell cultures compared to those of adults, generating an environment conceivably attracting the immigration of blood born precursors and promoting the propagation of skin-resident leukocytes (Schuster et al., 2009). This changing cytokine microenvironment within the developing skin could also be a reason for the gradual acquisition of RANK in our studies. We found that RANK expression besides cells of the periderm was restricted to HLA-DR^+^ epidermal leukocytes at every gestational step. The observations that LC precursors gradually augment the intensity of RANK expression with increasing gestational age and that leukocytes in prenatal dermis never express this marker lead us to speculate that they acquire this marker in the epidermis. These results confirmed our previous findings showing that in foreskins from infants and children the percentage of CD1a^+^RANK^−^ epidermal cells is higher compared to adult foreskins (Akgün, 2010). In conclusion, our data provide further evidence that besides HLA class II, CD1c, CD207 and CD1a also RANK/CD265 expression is acquired in LC precursors upon their arrival in the microenvironment of the skin.

It has been described that the secretion of the immunosuppressive cytokine IL-10 in gut DCs is initiated by RANK and that RANK/RANKL interaction leads to oral tolerance (Summers deLuca and Gommerman, 2012). Furthermore, the numbers and the function of T_reg_ cells during inflammation is regulated by RANK signaling of LCs (Loser and Beissert, 2007). Various studies have shown that the immune system in the human fetus is actively suppressed by a high number of T_reg_ cells in the circulation (Michaelsson et al., 2006 Tilburgs et al., 2008). In addition, high levels of IL-10 can be found in amniotic fluid (Cadet et al., 1995). These findings lead us to speculate that the interaction of RANK and its ligand could be involved in the IL-10 secretion by LC precursors in developing skin. It has been suggested that this autocrine IL-10 secretion may prevent the LC precursor emigration out of the skin and keep them in place. In addition, evidence exists that IL-10 is a key factor to maintain tolerance during pregnancy (Elbe-Bürger and Schuster, 2010).

Collectively, these data show that similar to other well known and important LC markers, RANK is acquired in LCs in a gradual manner during skin development and provide further important insight into human skin DC biology during gestation.

## Conclusion

This study provides further evidence for the concept that already in the prenatal skin epidermal leukocytes acquire markers that are essential for the protection of the unborn.

## Conflict of interest

None.
